# Genetic polymorphisms in the promoter region of catalase gene, creates new potential PAX-6 and STAT4 response elements

**Published:** 2016-06

**Authors:** Khyber Saify

**Affiliations:** Department of Biology, College of Sciences, and College of Stomatology, Kunduz University, Kunduz, Afghanistan

**Keywords:** Catalase, PAX-6, STAT4, Transcription factors

## Abstract

Catalase (*CAT*, OMIM: 115500) is an endogenous antioxidant enzyme and genetic variations in the regulatory regions of the *CAT *gene may alter the CAT enzyme activity and subsequently may alter the risk of oxidative stress related disease. In this study, potential influence(s) of the A-21T (rs7943316) and C-262T (rs1001179) genetic polymorphisms in the *CAT *promoter region, using the ALGGEN-PROMO.v8.3 online software were analyzed. Our findings show that the A allele at the -21 position creates a new potential binding site for PAX-6 and the T allele at the -262 position changes the TFII-I binding site into STAT4 response element. The PAX-6 and STAT4 are the multifunctional and enhancing transcription factors.

## INTRODUCTION

Oxidative stress may play a significant role in the risk of chronic diseases [1]. Although reactive oxygen species can cause oxidative damage to cellular macromolecules, such as DNA and lipids, multiple antioxidant defenses can neutralize reactive oxygen species [2].

Catalase (*CAT*, OMIM; 115500), an endogenous antioxidant enzyme, plays a major role in controlling hydrogen peroxide concentration in human cells. It decomposes H2O2 into H2O and O2, thereby protecting the cells from oxidative stress. It has been suggested that functional polymorphism in the gene encoding catalase enzyme affects the enzyme activity, thereby altering the protection against oxidative stress [3].

Several epidemiologic studies have suggested that single nucleotide polymorphism in *CAT *gene may be associated with many diseases, such as hypertension, cancers, diabetes, nephropathy, and other diseases accompanied by oxidative stress [3-9]. The polymorphisms such as C-262T (rs1001179) and A-21T (rs7943316) which are located in the promoter region of the *CAT *gene were found to be associated with altered catalase activity [10, 11]. In this study, we analyzed the A-21T and C-262T polymorphisms in the promoter region and their effects on transcription factor binding sites.

## MATERIALS AND METHODS

First, the catalase gene promoter sequence was obtained from the NCBI. Then both polymorphic sites (rs1001179) and (rs7943316) were identified on sequence. For identification of transcription factor binding site the ALGGEN-PROMO.v8.3 online software (http://alggen.lsi.upc.es/cgibin/promo_v3/promo/promoinit.cgi?dirDB=TF_ 8.3) was used. To identify transcription factor area, wild type allele and variant alleles were analyzed separately. In all analysis the maximum matrix dissimilarity rate was assumed 3%.

## RESULTS AND DISCUSSION

**Figure 1:**
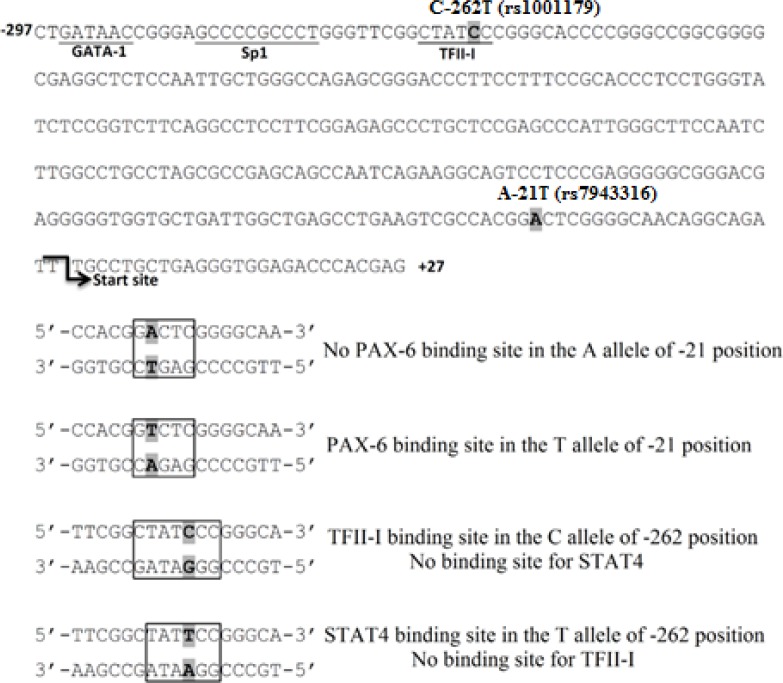
Genetic polymorphisms in the promoter region of the human catalase (*CAT*) gene and its influence on transcription factor recognition sites

The result shows that the -21A>T substitution in the promoter region of *CAT*, creates a new potential PAX-6 responsive element (G**A**C**A**C to G**T**CTC) (Fig. 1). Moreover, the C allele at the position -262 changes the TFII-I binding site (CTAT**C**CC) into STAT4 (CTAT**T**CC) responsive element (Fig. 1).

This is the first report showing that the A-21T and C-262T polymorphisms create new putative PAX-6 and STAT4 binding sites, respectively. The PAX-6 is a highly conserved multifunctional transcription factor and has been proposed to bind to promoter sequences of catalase gene. Also PAX-6 is a key regulatory gene of eye and brain development [12]. Previous study showed that the A allele of the *CAT *at the -844 position (rs769214) might create a binding site for PAX-6 [13]. The A allele on -844 position is the more frequent allele in Caucasian population and induced a higher transcriptional activity than the alternative allele (G allele) [13].

Our present findings also show that PAX-6 bind to the *CAT *promoter only if the T allele was present at the -21 positions. To our knowledge, the T allele at the -21 position may induce the *CAT *promoter activity. The signal transducer and activator of transcription (STAT) family of molecules is localized to the cytoplasm. STAT4 regulates various genes expression as a transcription factor and is involved in T helper cell (Th1) cell development [14].

The present results show that STAT4 binds to the *CAT *promoter only if the T allele is present at the -262 position; however the C allele is the putative binding site for TFII- 1 transcription factor. Oxidative stress induces catalase activity. In addition, studies have shown that reactive oxygen species activate the STAT family protein via serine/threonine kinases [15]. It may conclude that STAT4 may induce the *CAT *promoter activity. The influence of the *CAT *A-21T and C-262T polymorphisms on the *CAT *mRNA levels should be further researched.
